# Methodology and Reporting Quality Evaluation of Acupuncture for Mild Cognitive Impairment: An Overview of Systematic Reviews

**DOI:** 10.1155/2020/7908067

**Published:** 2020-08-05

**Authors:** Tinghui Hou, Qianhua Zheng, Xiumei Feng, Lu Wang, Ying Liu, Ying Li

**Affiliations:** ^1^Acupuncture and Tuina School, The Third Teaching Hospital, Chengdu University of Traditional Chinese Medicine, Chengdu, Sichuan, China; ^2^Graduate School, Chengdu University of Traditional Chinese Medicine, Chengdu, Sichuan, China

## Abstract

**Objective:**

Since there is no consistent evidence on the effectiveness of acupuncture in the treatment of mild cognitive impairment, this review aims to summarize and critically evaluate the methodological and reporting quality of systematic reviews (SRs).

**Methods:**

We comprehensively searched PubMed, Embase, Cochrane Library, Web of Science, China National Knowledge Infrastructure (CNKI), China Science and Technology Journal Database (VIP), Chinese Biomedical Literature (CBM), and Wanfang databases from the date of establishment to April 2019. Two authors independently selected the articles, collected the data, and assessed the identified and included SRs with the revised measurement tool to assess systematic reviews (AMSTAR 2) and preferred reporting items for SRs and meta-analyses (PRISMA). The quality of outcomes was evaluated by the Grading of Recommendations, Assessment, Development, and Evaluation (GRADE).

**Results:**

Eleven SRs were included in this overview. The items of AMSTAR 2 in most SRs were poorly reported; only 3 SRs were rated as low quality by AMSTAR 2, and the remaining were rated as very low quality. A total of 8 SRs obtained a decent rating by PRISMA. With the GRADE tool, we have not found high-quality evidence that acupuncture is effective for mild cognitive impairment (MCI), so there is no certain conclusion on the effectiveness of acupuncture treatment for MCI.

**Conclusion:**

The methodological and reporting quality of SRs on acupuncture for MCI is substandard, and the quality of evidence is poor. In future research, more efforts are needed to improve the quality of SRs in this field.

## 1. Introduction

Mild cognitive impairment (MCI) refers to mild impairment in one or more cognitive areas, but individuals are still independent and normal in their daily activities. It is a syndrome of cognitive impairment between normal ageing and Alzheimer's disease. It is mainly manifested by mild impairment of cognitive function that does not correspond to age, and the most typical symptom is memory loss [1, 2]. MCI can be divided into two types according to the different manifestations of cognitive impairment: amnestic MCI (aMCI) and nonamnestic MCI (NaMCI) [3]. NaMCI mainly affects cognitive areas such as judgement, decision-making ability, and visual perception. Memory shows relatively little impairment. In contrast, aMCI mainly affects memory, leaving other cognitive areas remain relatively intact, and this is the most common subtype of MCI [4]. Epidemiological studies have indicated that the incidence of MCI varies from 3% to 42% due to differences in diagnostic criteria and regions, with an estimated annual incidence rate of 21.5–71.3/1000 [5–9]. The prevalence of MCI increases with age [10, 11], and 5–15% of patients with MCI will develop dementia [12, 13].

The most common methods of diagnosing MCI in the clinic are subjective assessment and objective assessment [14]. Subjective assessment refers to the doctor inquiring about the patient's medical history and examining the patient's mental state. The objective assessment refers to relying on mental state examination scales [15]. Among them, the Mini-Mental State Examination (MMSE), [16] the Montreal Cognitive Assessment (MoCA), [17] the activity of daily life scale (ADL) [18], and the Mini-Cog text [19] are frequently used. There are several theories about the underlying causes of MCI, and accurately identifying the major underlying causes remains a challenge [20]. It is generally accepted that MCI is associated with Alzheimer's disease (AD) pathology, Lewy body pathology, cardiovascular and cerebrovascular diseases, and frontotemporal lobe degeneration [20–24]. There are no specific drugs recommended for the treatment of MCI [25]. Many drugs approved for the treatment of AD have been evaluated as potential therapeutic agents for MCI in many clinical trials, and these include cholinesterase inhibitors and nicotinic cholinergic receptors. In addition, vitamin B, vitamin E, and omega-3 fatty acids have also been proven effective for the treatment of MCI [20, 26, 27]. There is evidence that cognitive training and physical exercise are beneficial to MCI patients as nondrug interventions [3]. Acupuncture, as a traditional Chinese medicine therapy, has been increasingly developed and popularized in Western medicine. As one of the most popular complementary alternative therapies, it is widely used in clinical practice. In recent years, a large number of studies have shown that acupuncture treatment for MCI has significant clinical effectiveness [28, 29], can effectively delay the conversion of MCI to dementia, and can contribute to the early prevention and treatment of senile dementia [26].

Since the development of evidence-based medicine, high-quality systematic evaluation and meta-analysis have been considered the best evidence for evaluating randomized controlled trials [30]. Some remarkable results have been obtained in evaluating systematic reviews (SRs) of MCI patients treated with acupuncture and moxibustion, while others have failed to prove their effectiveness. These contradictory results can mislead clinicians, guideline makers, and other decision makers. Moreover, the quality of systematic evaluation can be degraded by factors such as design flaws, limitations of research, and publication bias [31]. Therefore, it is very important to evaluate the quality of SRs. At present, there are no relevant re-evaluation articles on the methodological quality and reliability of outcome indicators of systematic evaluations of acupuncture treatment for MCI.

Therefore, the main purpose of our study was to evaluate the SR methodology and reporting quality of acupuncture treatment for MCI to provide useful evidence for the clinical application of technology evaluated in SRs in the treatment of MCI patients.

## 2. Methods

### 2.1. Search Strategy

Systematic literature searches were conducted in PubMed, Embase, Cochrane Library, Web of Science, China National Knowledge Infrastructure (CNKI), China Science and Technology Journal Database (VIP), Chinese Biomedical Literature Database (CBM), and Wanfang database for SRs of MCI treated by acupuncture. All online databases were searched from their inception dates until April 22, 2019. The search terms used were (acupuncture or scalp acupuncture or electro-acupuncture), (MCI or Benign senescent forgetfulness or Age-related cognitive decline or Cognitive impairment-no dementia or Mild cognitive decline or Age-associated memory impairment or Forgetfulness or Hypomnesia or Memory decline), and (System evaluation or Systematic review or Meta-analysis), with minor modifications to individual searches in each database. In addition, the bibliographies of these papers, conference papers, and grey literature and published journal bibliographies were also retrieved. The retrieval language was limited to English and Chinese based on the researchers' languages. Two investigators (LY and FXM) independently searched the literature, and if they met with disagreements, they resolved them through discussion, see Tables 1–8 for detailed search strategies.

### 2.2. Study Selection

The criteria for selecting SRs were as follows: (1) RCTs or quasi-RCTs (q-RCTs) must be included in the SRs; (2) the subjects of the study were patients with definite diagnosis of MCI, and there were no restrictions on age, race, sex, and region; (3) the diagnosis of MCI should be based on the following criteria: one of the editions of the Diagnostic and Statistical Manual of Mental Disorders (DSM-III, DSM-IV, or DSM-V), Petersen diagnostic criteria, revised Mayo Clinic criteria, or Chinese Classification of Mental Disorders (CCMD-2 or CCMD-3); (4) the experimental group should be treated by acupuncture (manual acupuncture, scalp acupuncture, electroacupuncture, etc.) or by acupuncture combined with other therapies (traditional Chinese medicine/Chinese patent medicine, Western medicine, cognitive function training, etc.); (5) the control group was placebo therapy (blank control, sham acupuncture, sham acupoints, etc.) or other therapies; (6) one or more of the following evaluation methods were used: MMSE, MoCA, Mini-Cog test, Clinical Dementia Rating (CDR) scale, clock drawing test (CDT), ADL scale, and other assessment methods such as effectiveness; (7) studies involving cognitive impairment after stroke, vascular dementia, AD, senile dementia, and MCI caused by other diseases were excluded; (8) duplicate published studies were eliminated, and the highest-quality articles were included; and (9) papers were excluded if they were animal experiments, clinical RCT trials, conference literature, literature on nonmajor interventions of acupuncture, papers with data that could not be extracted, or bibliometrics research.

### 2.3. Data Extraction

Two researchers independently screened and extracted the literature following the inclusion and exclusion criteria. A unified document extraction form was formulated, which included title, first author, publication year, country, document type, retrieval database, retrieval terms, whether to retrieve registration platform, language restriction, included document type, whether to register or research programme has been published, research population, age, research sample size, treatment group intervention measures, control group intervention measures, methodological quality assessment tools, outcome indicators, main conclusions, subgroup analysis or sensitivity analysis, acupoint analysis or acupoint selection rules, safety adverse events, and whether RCTs in the included SRs strictly abide by STRICTA or CONSORT declaration. The data were independently extracted by two researchers (LY and FXM) and then cross-checked. If there was a disagreement, they were discussed or a third party (ZQH) was invited to evaluate them.

### 2.4. Quality Assessment

Three evaluation tools, AMSTAR 2, PRISMA, and GRADE, were used to evaluate the included SRs. AMSTAR 2 is a measurement tool used to evaluate the quality of the methodology of an SR [32]. PRISMA is a widely used reporting guide for SRs that provides an overall assessment of the quality of reports for systematic evaluation and meta-analysis [33]. GRADE tool is applied to classify the evidence quality of the main outcome indicators [34]. Two reviewers (FXM and WL) independently evaluated the incorporated SRs and resolved differences through discussion and negotiation. Any unresolved differences were judged by the third reviewer (ZQH).

#### 2.4.1. AMSTAR 2 for Methodological Quality

AMSTAR 2 is a recently updated rating scale that uses a rating process based on key area assessments. It has 16 questions, requiring respondents to answer “yes,” “no,” or “partially yes;” among them, 7 key items can be used to evaluate the effectiveness of the results, which are items 2, 4, 7, 9, 11, 13, and 15, and the remainder are nonkey items. The quality level of an SR is divided into four levels: none or only one nonkey item does not conform to the “advanced” level; more than one nonkey item do not conform to the “intermediate” level; only one key item does not conform to the criteria, with or without nonkey items that do not conform to the criteria of “low level,” and more than one key item does not conform, with or without nonkey items that do not conform to the “very low level.” Another practical way to use AMSTAR 2 to summarize methodological evaluation results is to calculate the percentage of entries that received a “yes” answer. In particular, the following categorization was used: >80% of the items receiving “yes” was considered fully reported; >50% of the items receiving “yes” was considered to have a good overall rating; and <50% of the items receiving “yes” was considered inadequate. The above calculation method with the percentage of “yes” items was also applicable to the PRISMA evaluation [35–38].

#### 2.4.2. PRISMA for Reporting Quality

The PRISMA [33] statement was used to evaluate the quality of reports, which included 27 items. According to the satisfaction degree of item reporting requirements, it can be divided into “yes,” “partial yes,” and “no.” The completion of each item was presented as a ratio.

#### 2.4.3. GRADE for Quality of Evidence

The GRADE tool evaluates the quality of evidence for the main outcome indicators of the included SRs [34]. Factors leading to RCT degradation include research limitations, inconsistency of research results, inability to determine whether it is direct evidence, insufficient accuracy or wide confidence intervals, and publication bias. Its evaluation method is as follows: no evidence of degradation is high quality, 1 degradation is moderate quality, 2 degradations are low quality, and 3 degradations or more are very low quality.

### 2.5. Data Analysis

We used SPSS 20.0 (IBM Corp, Armonk, NY, USA) for statistical analysis when necessary. The significance level of all analyses was 0.05 for a bilateral test. Summary statistics are presented in terms of frequency and percentage. We used the kappa index to calculate the reliability between two evaluators using the AMSTAR 2 and PRISMA scales. A kappa index greater than 0.75 reflected excellent consistency; 0.4 to less than 0.75 indicated fair consistency; and less than 0.4 indicated poor consistency.

## 3. Results

### 3.1. Selection of Systematic Reviews

**Figure 1:**
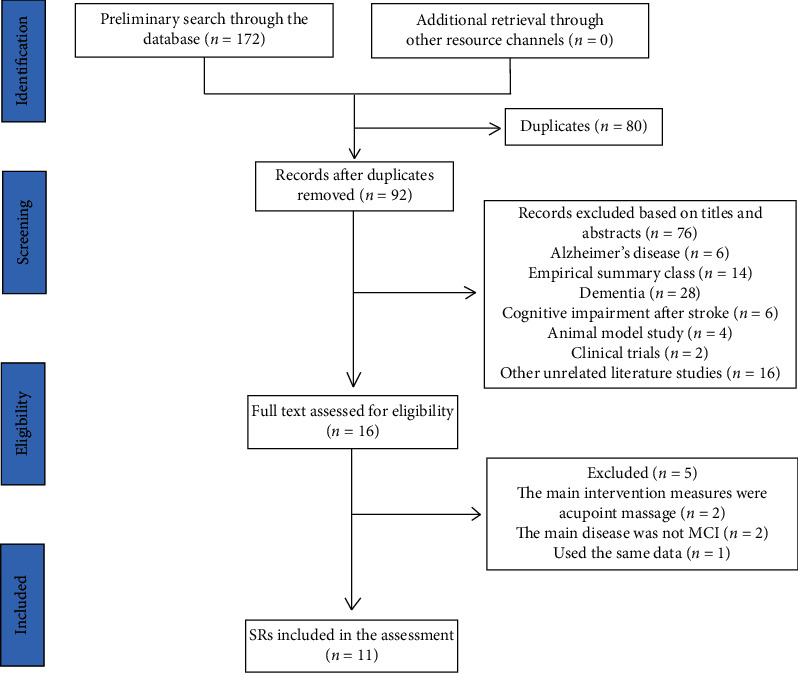
Flowchart of literature selection.

A total of 172 potentially related kinds of literature were retrieved from 8 databases, including 3 from Cochrane Library, 21 from Embase, 15 from PubMed, 7 from Web of Science, 36 from CNKI, 27 from CBM, 17 from VIP, and 46 from the Wanfang database. After 80 duplicated documents were deleted by EndNote X9 software (Thomson ResearchSoft, Stanford, CT, USA) and the 92 titles and abstracts of the remaining documents were read, 76 documents were excluded because they failed to meet the inclusion criteria. After downloading and reading the other 16 articles, 4 studies were excluded. The reasons for exclusion were as follows: the main intervention measures of two articles were acupoint massage, and the main diseases examined in two articles were not MCI. Detailed information on the excluded studies can be found in Table 9. Finally, our study included 11 studies [39–49]. The process of the literature search and screening is shown in Figure 1.

### 3.2. Characteristics of the Systematic Reviews

In terms of searching the literature bases, 3 SRs [41, 45, 49] were identified in Embase; 4 SRs [41, 42, 45, 49] in PubMed; 3 SRs [42, 45, 49] in Web of Science; 5 SRs [39, 40, 43, 46, 48] in Wanfang; 4 SRs [40, 43, 44, 48] in CBM; 3 SRs [44, 47, 48] in CNKI; and 3 SRs [43, 44, 48] in VIP. Six SRs [39, 40, 43, 44, 47, 48] were published in Chinese journals, and the other 5 [41, 42, 45, 46, 49] were published in English journals. Our research included 9 peer-reviewed articles [40–46, 48, 49] and 2 Master's theses [39, 47]. Documents were mostly published between 2011 and 2019, with 10 SRs [39–48] from China and 1 SR [49] from Korea. The minimum and maximum numbers of included studies in the SRs were 5 and 25, respectively. The minimum number of MCI cases was 232, and the maximum number was 1847. In terms of intervention measures, 6 studies [41–44, 48, 49] involved treatment with acupuncture alone, and 5 studies [39, 40, 45–47] involved treatment with acupuncture combined with other therapies. The control group received treatments including no acupuncture, ordinary acupuncture, Chinese patent medicine, Western medicine, and cognitive function training. The main outcome indicators used several different evaluation scales, and the most commonly used were the MMSE and MoCA. Other indicators include efficacy, apparent efficacy, Wechsler Memory Scale (WMS), Clinical Memory Scale (CMS), CDT, modified Barthel Index (MBI), ADL scale, and Memory Quotient (MQ). Methodological evaluation tools were used in most of these SR studies. The Cochrane Manual was used in 8 studies [39, 42–46, 48, 49], the CONSORT and STRICTA guidelines were used in 1 study [41], and the other 2 studies [40, 47] did not mention methodological evaluation tools. Subgroup analyses were mentioned in 9 studies [39, 42–49], and acupoint selection was mentioned in 3 studies [45, 47, 49]. There was no agreement on the effectiveness of acupuncture among all the SRs included. Detailed basic features are shown in Table 10.

### 3.3. Quality of the Systematic Reviews

#### 3.3.1. Methodological Quality of Included Reviews (AMSTAR 2)

**Figure 2:**
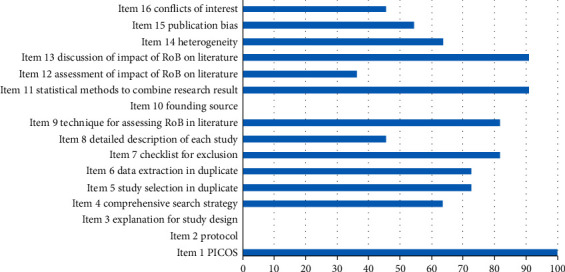
Percentage of studies with “Yes” for each AMSTAR 2 item.

There was no SR with AMSTAR 2 scores at the high and intermediate levels; only 3 SRs (27.27%) [43, 46, 48] scored at low overall levels, while the other 8 SRs (72.73%) [39–42, 44, 45, 47, 49] scored at very low levels. The 11 SRs (100.00%) contained the population, intervention, comparator group, outcome, and study design (PICOS) components. No SR formulated a preliminary research protocol before making a systematic evaluation. No SR explained the design types included in the study. Seven SRs (63.64%) achieved comprehensive literature retrieval. Three SRs (27.27%) did not describe the repeatability of research screening and data extraction. Nine SRs (81.82%) provided a list of excluded studies or justification for their exclusion. Only 5 SRs (45.45%) provided details of the basic information included in the studies. The bias risk assessment methods of 9 SRs (81.82%) were reasonable. No SR reported funding information for the studies they included. Ten SRs (90.91%) used appropriate statistical methods to synthesize the results. Only 4 SRs (36.36%) assessed the impact of bias risk on meta-analysis results. Ten SRs (90.91%) considered the bias risk of individual studies when interpreting and discussing the results. When heterogeneity was observed in the review results, 7 SRs (63.64%) investigated the sources of heterogeneity and discussed its impact on the review results. Six SRs (54.55%) fully investigated the possibility of publication bias and discussed the impact of publication bias on outcomes when quantitatively merging the results. Five SRs (45.45%) reported that the SR itself had no competitive conflicts of interest, four of which reported funding sources. Of all the projects, five of the projects were fully reported, including PICO construction, excluded literature lists with reasons, reasonable bias risk assessment methods, appropriate statistical analysis methods, and the bias risk of a single study which was taken into account when interpreting and discussing the results of systematic evaluation. Nevertheless, there were still six bad items, including the prior protocol, description of the type of study included, detailed basic information, funding information for included research, the impact of single research bias risk on meta-analysis results, and sources of conflicts of interest, see Table 11 and Figure 2 for details. The interrater reliability was excellent (kappa = 0.859) between the two assessors (FXM and WL). Disagreements were found in some domains after comparison, and more details are shown in Tables 12 and 13. Finally, disagreements were settled by discussion and by input from a third author (ZQH).

#### 3.3.2. Reporting Quality of Included Reviews (PRISMA)

**Figure 3:**
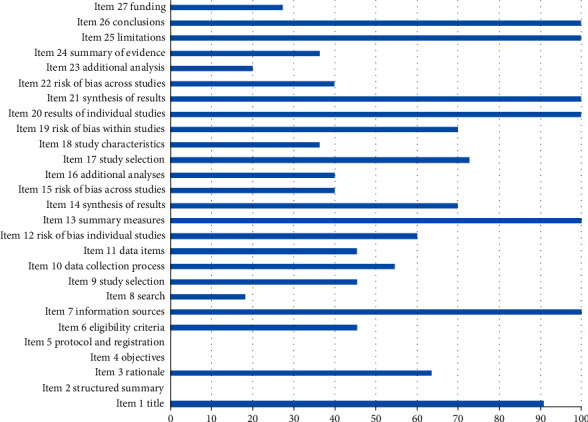
Percentage of studies with “Yes” for each PRISMA item.

None of the SRs completed all 27 items of PRISMA, but a total of 8 SRs [39, 41–43, 45, 46, 48, 49] scored well in PRISMA. Ten SRs (90.91%) explicitly mentioned the term systematic review or meta-analysis in the title. No SRs fully provided highly structured abstraction and protocol registration information. Seven SRs (63.64%) described the basic principles of review. No SR explicitly addressed the issue of PICOS. No SR provided the protocol and registration number in the methods section. Five SRs (45.45%) were carefully and completely reported according to the established eligibility criteria. All SRs (100.00%) described all sources of information in the search. Only 2 SRs (18.18%) provided an all-electronic search strategy for at least one major database. Five SRs (45.45%) explained the procedure of the selection study. Six SRs (54.55%) described the process of data acquisition. Five SRs (45.45%) listed and defined all variables that required data. Except for Lu et al. [41], 6 SRs (60.00%) described methods for assessing bias risk in individual studies, all SRs (100.00%) described the main general indicators, 7 SRs (70.00%) described methods of processing data and merging research results, 4 SRs (40.00%) described any bias risk that might affect cumulative evidence, and 4 SRs (40.00%) described methods of additional analysis. In the results section, 8 SRs (72.73%) described the number of studies selected for screening, evaluation of eligibility, and inclusion in the review. Four SRs (36.36%) described the characteristics of the included studies. With the exception of Xiao et al. [41], 7 SRs (70.00%) provided bias risk data for each study, all SRs (100.00%) considered all the results of each study and provided composite results, 4 SRs (40.00%) submitted the assessment results of bias risk for all studies, and 2 SRs (20.00%) performed additional analyses. In the discussion section, 4 SRs (36.36%) summarized the main results evidently and generally. All SRs (100.00%) discussed the limitations of the research and results and provided general explanations of the results in the context of other evidence, as well as implications for future research. Three SRs (27.27%) provided information about the sources of funding and the role of funders. Overall, seven projects were fully reported, including the title, information sources, general measures, results of individual studies, result synthesis, limitations, and conclusions. However, there are still 14 items that were poorly reported, including structured summaries, objectives, protocols and registrations, eligibility criteria, search strategies, research selection procedures, data items, methodological aspects of the research bias risk, additional analysis, research characteristics of the results section, results of additional analysis, summary of evidence for discussion, and funding. The relevant results can be found in Table 14 and Figure 3. The interrater reliability was excellent between the two assessors (FXM and WL) (kappa = 0.929). Disagreements were found in some items after comparison, and more details are shown in Tables 15 and 16. Finally, discrepancies were resolved by discussion and by consulting a third author (ZQH).

#### 3.3.3. Quality of Evidence in Included Reviews (GRADE)

In the current study, with the exception of Liu et al. [40] and Lu et al. [41], the 9 SRs included 31 outcome indicators related to acupuncture treatment of MCI. The results of GRADE evaluation showed that 4 (12.9%) outcomes were of moderate quality, 8 (25.8%) were of low quality, and 19 (61.3%) were very low. The limitations of all results have been downgraded, and they were biased in randomness, distribution, concealment, and blindness followed by publication bias (*n* = 23, 74.2%), imprecision (*n* = 15, 48.4%), inconsistency (*n* = 14, 45.2%), and indirectness (*n* = 0, 0.0%). The relevant results can be found in Table 17. The interrater reliability was general between the two assessors (FXM and WL) (kappa = 0.644). Disagreements were found in some items after comparison, and more details are shown in Tables 18 and 19. Finally, discrepancies were resolved by discussion and by consulting a third author (ZQH).

### 3.4. Effects of Acupuncture

In the current study, with the exception of Liu et al. [40] and Lu et al. [41], all the other SRs were analysed by subgroup. Since the obtained data could not be quantitatively analysed, a descriptive analysis method was adopted to evaluate the relevant outcome indicators of the meta-analysis in the studies included in this study. Several analysis situations are summarized as follows.

#### 3.4.1. MMSE Scores

**Figure 4:**
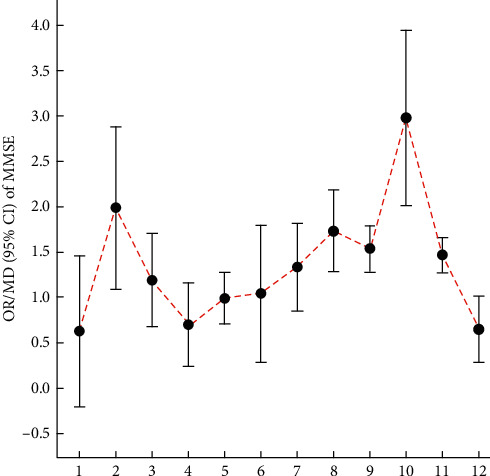
Comparison of the OR/MD value (95% CI) of the MMSE in included SRs.

Mao [39] found no difference between the therapeutic effect of acupuncture and donepezil hydrochloride on MCI (MD: 0.63, 95% CI: −0.20 to 1.46, *P* = 0.14; 2 trials). Cao et al. [42] suggested that acupuncture (MD: 1.99, 95% CI: 1.09–2.88, *P* < 0.0001; 6 trials) in conjunction with CFT/donepezil (0.25 mg daily) improved MMSE scores compared with CFT/donepezil alone. Hu et al. [43] found that there was a statistically significant difference between acupuncture combined with nimodipine (OR: 1.19, 95% CI: 0.67–1.70, *P* < 0.00001; 6 trials) or donepezil (OR: 0.70, 95% CI: 0.24–1.17, *P* = 0.003; 2 trials) in the treatment of MCI compared with nimodipine or donepezil alone. Deng and Wang [45] inferred that acupuncture significantly improved MMSE scores relative to the nimodipine group (MD: 0.99, 95% CI: 0.71–1.28, *P* < 0.01; 3 trials), and acupuncture combined with nimodipine significantly improved MMSE scores compared with nimodipine therapy alone (MD: 1.09, 95% CI: 0.29–1.89, *P* < 0.01; 2 trials). Mai and Zheng [44] found a significant difference in the treatment of MCI with scalp and electroacupuncture compared with nimodipine in improving MMSE scores (MD: 1.33, 95% CI: 0.85–1.82, *P* < 0.00001; 3 trials). Ting et al. [46] suggested that acupuncture plus drug for MCI could significantly improve MMSE scores (MD: 1.73, 95% CI: 1.28–2.18, *P* < 0.00001; 12 trials). Wang [47] pointed out that acupuncture or acupuncture combined with other therapies compared with medication for MCI has advantages in improving MMSE scores (MD: 1.54, 95% CI: 1.29–1.80, *P* < 0.00001; 7 trials). In addition, she suggested that the MMSE scores with the experience of acupuncture for MCI were better than those with ordinary acupuncture (MD: 2.98, 95% CI: 2.01–3.95, *P* < 0.00001; 3 trials). Li et al. [48] demonstrated that the MMSE scores in the experimental group showed greater improvement than those in the control group (MD: 1.47, 95% CI: 1.27–1.66, *P* < 0.00001; 9 trials). Kim et al. [49] suggested that there was a significant difference (MD: 0.65, 95% CI: 0.28–1.01, *P* = 0.0005; 6 trials) between the electroacupuncture and syndrome differentiation group or electroacupuncture alone group versus the antidementia drug group. The relevant results can be found in Table 20 and Figure 4.

#### 3.4.2. MoCA Scores

**Figure 5:**
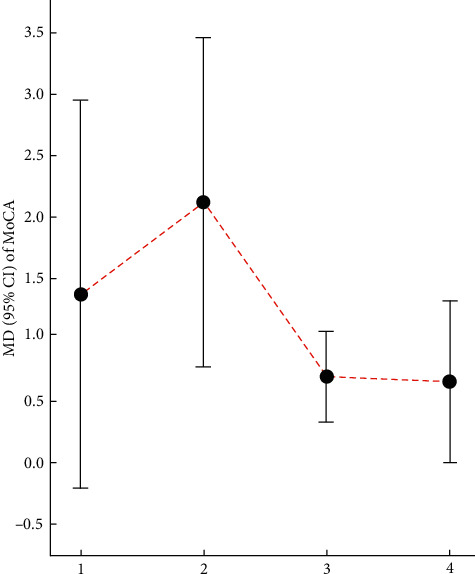
Comparison of the MD value (95% CI) of MoCA in included SRs.

Cao et al. [42] found that there was no significant difference between electroacupuncture in combination with donepezil and donepezil alone (MD: 1.37, 95% CI: −0.21–2.95, *P* = 0.09; 1 trial). Mai and Zheng [44] suggested that scalp acupuncture combined with CFT for MCI significantly improved the MoCA scores compared with CFT (MD: 2.12, 95% CI: 0.78–3.47, *P* = 0.002; 2 trials). Li et al. [48] found that the MoCA scores in the test group showed greater improvement than those in the control group in the treatment of MCI (MD: 0.70, 95% CI: 0.33–1.07, *P* = 0.0002; 5 trials). Kim et al. [49] suggested that electroacupuncture was equivalent to an antidementia drug (MD: 0.66, 95% CI: 0.00–1.32, *P* = 0.05; 2 trials). The relevant results can be found in Table 21 and Figure 5.

#### 3.4.3. CMS Scores

Mao [39] found that there was no significant difference in the efficacy of either acupuncture alone or acupuncture combined with donepezil hydrochloride in the treatment of MCI compared with donepezil hydrochloride.

#### 3.4.4. MQ Scores

Mao [39] found no difference between the therapeutic effect of acupuncture on mild cognitive dysfunction and donepezil hydrochloride. The GRADE rating is very low level.

#### 3.4.5. ADL Scores

**Figure 6:**
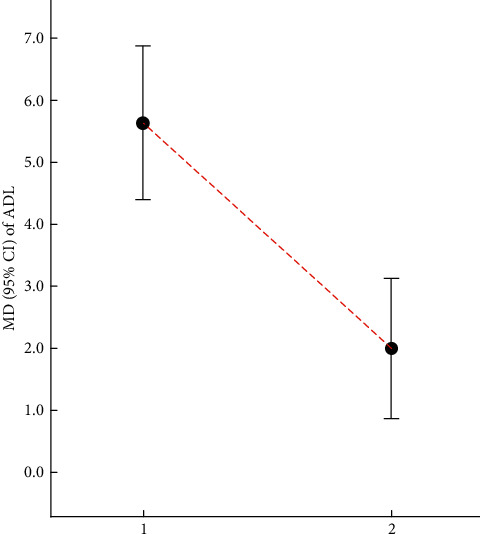
Comparison of the MD value (95% CI) of ADL in included SRs.

Ting et al. [46] indicated that the ADL scores could be significantly improved by acupuncture plus drug interventions (MD: 5.63, 95% CI: 4.40–6.87, *P* < 0.001; 6 trials). Li et al. [48] found that the improvement in the ADL scores in the test group was better than that in the control group in the treatment of MCI (MD: 2.00, 95% CI: 0.88–3.12, *P* = 0.0005; 3 trials). The relevant results can be found in Table 22 and Figure 6.

#### 3.4.6. CDT Scores

Li et al. [48] proposed that the CDT score improvement in the experimental group was better than that in the control group in the treatment of MCI (MD: 0.63, 95% CI: 0.47–0.79, *P* < 0.00001; 2 trials). The relevant results can be found in Table 23.

#### 3.4.7. MBI Scores

**Figure 7:**
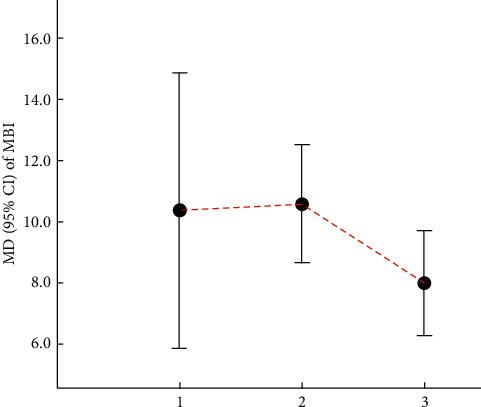
Comparison of the MD value (95% CI) of MBI in included SRs.

Cao et al. [42] indicated that electroacupuncture in combination with CFT was superior to CFT alone (MD: 10.73, 95% CI: 6.25–15.21, *P* < 0.00001; 1 trial), and electroacupuncture in conjunction with nimodipine (90 mg daily) was also superior to nimodipine (90 mg daily) alone (MD: 10.57, 95% CI: 8.64–12.50, *P* < 0.00001; 1 trial). In addition, they reported that electroacupuncture effectively increased MBI scores compared with nimodipine (90 mg daily) (MD: 7.99, 95% CI: 6.29–9.69, *P* < 0.0001; 1 trial). The relevant results can be found in Table 24 and Figure 7.

#### 3.4.8. Effective Rate

**Figure 8:**
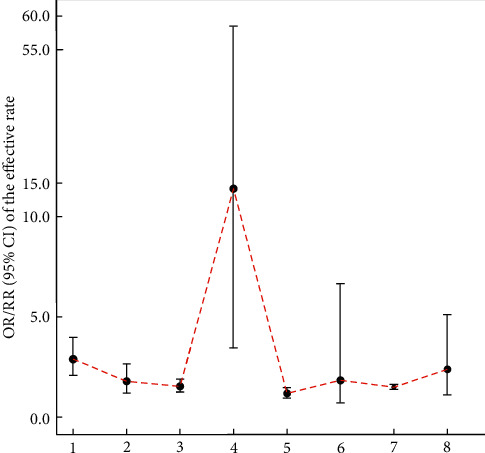
Comparison of the MD value (95% CI) of the effective rate in included SRs.

Hu et al. [43] suggested that acupuncture can increase the number of MCI patients showing an effective response (OR: 2.89, 95% CI: 2.10–3. 97, *P* < 0. 00001; 9 trials). Deng and Wang [45] demonstrated that the clinical efficacy rate was significantly higher in the acupuncture group than in the nimodipine group (OR: 1.78, 95% CI: 1.19–2.65, *P* < 0.01; 3 trials). Mai and Zheng [44] suggested that the total effective rate of scalp and electroacupuncture for MCI was better than that with nimodipine (RR: 1.53, 95% CI: 1.25–1.89, *P* < 0.0001; 3 trials), and the total effective rate of scalp acupuncture combined with CFT was equivalent to CFT (RR: 1.18, 95% CI: 0.94–1.47, *P* = 0.16; 2 trials). Mai and Zheng [44] also pointed out that the apparent efficacy of scalp and electroacupuncture for MCI was better than nimodipine (RR: 14.17, 95% CI: 3.44–58.44, *P* = 0.0002; 3 trials), and scalp acupuncture combined with CFT was equivalent to CFT in apparent efficiency (RR: 1.83, 95% CI: 0.72–6.64, *P* = 0.20; 2 trials). Wang [47] suggested that acupuncture treatment of MCI can increase the effective rate (RR: 1.49, 95% CI: 1.36–1.62, *P* < 0.0001; 16 trials). Li et al. [48] pointed out that the total effective rate for MCI in the experimental group was better than that in the control group (RR: 2.38, 95% CI: 1.11–5.11, *P* = 0.03; 2 trials). The relevant results can be found in Table 25 and Figure 8.

## 4. Adverse Reactions

A total of 8 SRs [39, 42–47, 49] mentioned adverse events, and the remaining 3 SRs did not mention them. Of these 8 SRs, 2 [42, 49] stated that there were no adverse events reported in the literature of the studies they included. The remaining studies mainly reported that the adverse events caused by acupuncture were pain [39], headache [44, 47], dizziness [44, 47], bleeding [44, 46], stun on acupuncture [44, 47], subcutaneous congestion [43], haematoma [44, 47], errhysis [45], fainting [45], diarrhoea [46], etc., and the adverse reactions caused by Western medicine included elevated transaminase [39], diarrhoea [46], and gastrointestinal reactions [45]. It can be seen that acupuncture will not cause important adverse events or even induce negative effects.

## 5. Discussion

### 5.1. The Methodological Quality of Systematic Evaluations of Acupuncture for MCI Is Not High

SRs are regarded as the highest quality evidence for guiding clinical decision-making [50]. With the emergence of an increasing number of studies on acupuncture treatment of MCI at home and abroad, the number of SRs in the literature evaluating the effectiveness of acupuncture is also increasing. However, deficiencies in research methodology and SR reporting often contribute to the reduced validity of conclusions. To our knowledge, this study is the first to evaluate the quality of the methodology and reporting of acupuncture for the treatment of MCI and to provide useful recommendations for future evaluators. Regrettably, the results of this study found that most SRs had problems in research methods and reporting quality, and the common problems were mainly reflected in the following aspects: (1) the systematic evaluation lacks the preliminary research programme; (2) the SR did not develop a detailed search strategy; (3) the SR did not provide a detailed list of excluded literature; (4) biased risk assessment was not accurate; (5) the influence of bias risk was not considered in the discussion and interpretation of the results; and (6) the conflicts of interest and funding sources were not reported. To some extent, this reflects the inconsistencies in the conclusion of the systematic evaluation and provides a reminder to improve the quality of SRs in future studies.

### 5.2. Acupuncture Can Improve MMSE Scores and Effective Rate in Patients with MCI, but the Quality of Evidence Is Not High

This study was originally intended to subdivide the subgroup analyses from different intervention methods of acupuncture on the basis of subgroup analysis through different scale scores. However, the intervention methods in most of the literature included in this study were not simple acupuncture. The combination methods were complex and diverse, and what kind of joint method was used was not adequately explained, so the subgroups could not be subdivided according to this assumption; therefore, different scales were used for this descriptive analysis. From this, we found that most literature studies indicated that acupuncture alone or in combination with other treatment methods increased MMSE scores and the effective rate, but there was still evidence that acupuncture would not increase them or may even produce the opposite result. Obviously, this reduces the utility of using the MMSE scale and the effective rate of acupuncture in patients with MCI. In addition, none of the literature explained whether patients had a basic acupuncture history before receiving acupuncture (such as acupuncture for diseases involving pain). Although most literature studies supported acupuncture for improving these clinical indicators, it was not clear whether other confounding factors, such as combined therapy, inappropriate control groups, cumulative acupuncture effects, or nonspecific effects of acupuncture, affected the results, and the reliability of the evaluation will decrease accordingly.

The other outcome indicators, such as the MoCA, CMS, MQ, ADL, MBI, and CDT, used in the results of the subgroup analysis, cannot be used to show that acupuncture therapy was superior to the control group due to the small number of studies described.

### 5.3. The Methodological Quality of SRs Was Assessed Using the AMSTAR 2 Tool, Which Was Stricter and More Comprehensive than AMSTAR

As a new evaluation tool released in 2017, AMSTAR 2 [32] was created by combining various opinions and undergoing strict revision procedures. Compared with AMSTAR, AMSTAR 2 refines the evaluation criteria for each item. It added and refined the evaluation items on the basis of the original scale and no longer adopts the total score method. It can be used to report the methodological quality of the systematic evaluation literature more directly and objectively, with good validity, reliability, and applicability. The main modifications included the evaluation of nonrandomized research in SRs, elaboration of PICO, treatment of risk of bias (RoB) in the process of evidence synthesis, possible reasons for heterogeneity and discussion of its impact, study of the rationality of design choices, and more. It was used for the first time to evaluate the methodology and reporting quality of acupuncture treatment for MCI. Researchers rigorously evaluated each item during use. Although the literature included in this study was evaluated by AMSTAR 2, the results showed that the quality of the vast majority of the literature is extremely low. At the same time, on the contrary, it indicated that the evaluation results are highly credible and have good consistency and practicality among the evaluators. Therefore, in future systematic reviews, AMSTAR 2 is recommended as a tool for evaluating method quality.

### 5.4. The Design of Acupuncture Clinical Studies Is a Challenge

The clinical design of studies with acupuncture has always been a challenge. No one can design a perfect and rigorous clinical scheme, especially methods for blinding of acupuncturists. Due to the different design methods used in each study, the bias caused by different blinding effects of physicians is unavoidable. In addition, most of the literature studies in this study did not mention the acupoints used, and only three SRs mentioned the acupoints used in the study. Mao [39] mentioned that GV20, GB20, GV24, and EX-HN1 were commonly used acupoints. Lu et al. [41] mentioned the top 8 acupoints most frequently used: GV20, EX-HN1, GV24, GB20, GB13, KI03, LR03, and ST36. Hu et al. [43] mentioned that the most commonly used acupoints were GV20, GB20, EX-HN1, GV24, KI03, BL23, GB39, LR03, ST40, SP10, ST36, GB13, BL18, PC06, LI04, KI06, and HT07. It can be seen that there is no unified and standard acupuncture point for acupuncture treatment of MCI, but it is mainly based on dialectical traditional Chinese medical theories such as tonifying the liver and kidney, resolving phlegm and resuscitation, and relieving the liver and qi and then applying the treatment. In addition, the course of acupuncture treatment for MCI cannot be unified, which had an inevitable impact on the evaluation of SRs.

### 5.5. Limitations

We must acknowledge the limitations in this study: (1) the quality defects of the included SRs are to a certain extent, a key factor affecting our evaluation results. (2) Only Chinese and English SRs were included. Language limitations may have affected the results. (3) This was the first time that the evaluation analysts used the AMSTAR 2 scale. The evaluation of many items involved is inevitably subjective and may have led to bias. (4) The interventions included in the systematic review were diverse and cannot be quantitatively combined to analyse their effect values.

## 6. Conclusion

Acupuncture, either alone or as an adjunct to other interventions, has been effectively applied in the clinical practice of treating patients with MCI. Based on the AMSTAR 2 and PRISMA guidelines, this study evaluated studies with poor methods and reporting of acupuncture for MCI. According to the GRADE quality evaluation results, this review provides low-quality evidence for the effectiveness of acupuncture in the treatment of MCI. Although we cannot draw clear conclusions due to the poor quality of the included SRs, the available evidence suggests that acupuncture may be effective for MCI to some extent. In the future, SRs and RCTs in this field should improve their methodology and reporting quality in accordance with strict design principles.

## Figures and Tables

**Table 1:** Search strategy in the PubMed database.

Item	Index terms
#1	Acupuncture [All fields]
#2	Scalp acupuncture [All fields]
#3	Electroacupuncture [All fields]
#4	#1 OR #2 OR #3
#5	Mild cognitive impairment [All fields]
#6	Benign seneslent forgetfulness [All fields]
#7	Age-related cognitive decline [All fields]
#8	Cognitive impairment-no dementia [All fields]
#9	Mild cognitive decline [All fields]
#10	Age-associated memory impairment [All fields]
#11	Forgetfulness [All fields]
#12	Hypomnesia [All fields]
#13	Memory decline [All fields]
#14	#5 OR #6 OR #7 OR #8 OR #9 OR #10 OR #11 OR #12 OR #13
#15	System evaluation [All fields]
#16	System review [All fields]
#17	Meta analysis [All fields]
#18	#15 OR #16 OR #17
#19	#4 AND #14 AND #18

**Table 2:** Search strategy in the Cochran Library database.

Item	Index terms
#1	Acupuncture [Ti, Ab, Kw]
#2	Scalp acupuncture [Ti, Ab, Kw]
#3	Electroacupuncture [Ti, Ab, Kw]
#4	#1 OR #2 OR #3
#5	Mild cognitive impairment [Ti, Ab, Kw]
#6	Benign seneslent forgetfulness [Ti, Ab, Kw]
#7	Age-related cognitive decline [Ti, Ab, Kw]
#8	Cognitive impairment-no dementia [Ti, Ab, Kw]
#9	Mild cognitive decline [Ti, Ab, Kw]
#10	Age-associated memory impairment [Ti, Ab, Kw]
#11	Forgetfulness [Ti, Ab, Kw]
#12	Hypomnesia [Ti, Ab, Kw]
#13	Memory decline [Ti, Ab, Kw]
#14	#5 OR #6 OR #7 OR #8 OR #9 OR #10 OR #11 OR #12 OR #13
#15	System evaluation [Ti, Ab, Kw]
#16	System review [Ti, Ab, Kw]
#17	Meta analysis [Ti, Ab, Kw]
#18	#15 OR #16 OR #17
#19	#4 AND #14 AND #18

Ti, Ab, Kw: title, abstract, and keyword.

**Table 3:** Search strategy in the Embase database.

Item	Index terms
#1	Acupuncture OR (scalp acupuncture) OR electroacupuncture [All fields]
#2	Mild cognitive impairment OR (benign seneslent forgetfulness) OR (age-related cognitive decline) OR (cognitive impairment-no dementia) OR (mild cognitive decline) OR (age-associated memory impairment) OR forgetfulness OR hypomnesia OR (memory decline) [All fields]
#3	System evaluation OR (system review) OR (meta analysis) [All fields]
#4	#1 AND #2 AND #3

**Table 4:** Search strategy in the Web of Science database.

Item	Index terms
#1	TS = (Acupuncture OR Scalp acupuncture OR Electroacupuncture)
#2	TS = (Mild cognitive impairment OR Benign seneslent forgetfulness OR Age-related cognitive decline OR Cognitive impairment-no dementia) OR Mild cognitive decline OR Age-associated memory impairment OR Forgetfulness OR Hypomnesia OR Memory decline)
#3	TS = (System evaluation OR System review) OR Meta analysis)
#4	#1 AND #2 AND #3

TS = topic search: search included title, abstract, author keywords, and Keywords Plus.

**Table 5:** Search strategy in the CNKI database.

Item	Index terms
#1	SU = (‘Acupuncture'+‘Scalp acupuncture'+‘Electroacupuncture') AND SU = (‘Mild cognitive impairment'+‘Mild cognitive decline'+‘Dementia'+‘Forgetfulness'+‘Amnesia'+‘Memory in decline'+‘Memory loss') AND SU = (‘System evaluation'+‘Meta analysis'+‘System review')

SU: subject search.

**Table 6:** Search strategy in the VIP database.

Item	Index terms
#1	U = (Acupuncture OR Scalp acupuncture OR Electroacupuncture) AND U = (Mild cognitive impairment OR Mild cognitive decline OR Dementia OR Forgetfulness OR Amnesia OR Memory in decline OR Memory loss) AND U = (System evaluation OR Meta analysis OR System review)

U: all fields.

**Table 7:** Search strategy in the Wanfang database.

Item	Index terms
#1	Subject: (“acupuncture” or “scalp acupuncture” or “electroacupuncture”) and Subject: (“Mild cognitive impairment” or “Mild cognitive decline” or “dementia” or “forgetfulness” or “Amnesia” or “Memory in decline” or “Memory loss”) and Subject: (“System evaluation” or “Meta analysis” or “System review”)

**Table 8:** Search strategy in the CBM database.

Item	Index terms
#1	(“Acupuncture”[Common fields: Intelligent retrieval] OR “scalp acupuncture”[Common fields: Intelligent retrieval] OR “Electroacupuncture”[Common fields: Intelligent retrieval])
#2	(“Mild cognitive impairment”[Common fields: Intelligent retrieval] OR “Mild cognitive decline”[Common fields: Intelligent retrieval] OR “Dementia”[Common fields: Intelligent retrieval] OR “Forgetfulness”[Common fields: Intelligent retrieval] OR “Amnesia”[Common fields: Intelligent retrieval] OR “Memory in decline”[Common fields: Intelligent retrieval] OR “Memory loss”[Common fields: Intelligent retrieval])
#3	(“System evaluation”[Common fields: Intelligent retrieval] OR “Meta analysis”[Common fields: Intelligent retrieval] OR “System review”[Common fields: Intelligent retrieval])
#4	#1 AND #2 AND #3

Adopt intelligent search: realizes the extended search of search words and their synonyms (including subject words).

**Table 9:** Excluded documents after reading the full text.

References	Reason for excluding references
Chan-Young K, Boram L, Hyo-Weon S, et al. Efficacy and Safety of Auricular Acupuncture for Cognitive Impairment and Dementia: A Systematic Review. Evid Based Complement Alternat Med. 2018: 3426078. DOI: 10.1155/2018/3426078.	The main research disease is not mild cognitive impairment
Cheng-Hwang P, Yue-Cune C, Ruu-Fen T, et al. The treatment of cognitive dysfunction in dementia: a Multiple treatments meta-analysis. Psychopharmacology, 2018: 235(5), 1571–1580. DOI:10.1007/s00213-018-4867-y.	The main research disease is not mild cognitive impairment
Fang L, Cuiling S, Liqun Y, et al. Acupoint Massage for Managing Cognitive Alterations in Older Adults: A Systematic Review and Meta-Analysis. The Journal of Alternative and Complementary Medicine, 2018:24(6), 532–540. DOI:10.1089/acm.2017.0142.	The main intervention is not acupuncture, but acupoint massage
Kaili S. Research on the Traditional Chinese Medicine Nursing Intervention of Mild Cognitive Impairment in Community[Master's thesis]. Hubei University of Traditional Chinese Medicine, 2018.	The main intervention is not acupuncture, but acupoint massage
Zhou L, Zhang YL, Cao HJ, et al. Systematic review of acupuncture for vascular-induced mild cognitive impairment [J]. Chinese Journal of Integrated Traditional Chinese and Western Medicine. 2013; 33(12):1626–1630.	The data are the same as those of Cao Hui Juan's paper

**Table 10:** Characteristics of systematic reviews.

Study	Country	No. of primary studies	Study types	Intervention		Primary outcomes	Methodological evaluation tool	Subgroup analysis	Acupuncture point analysis	Main conclusions
				Treatment group	Control group					
Mao [39]	China	5 (232)	RCT or q-RCT	Acupuncture alone or combined with other treatments	Donepezil hydrochloride or the same basic treatment as the treatment group	MMSE, MQ, CMS	Cochrane Handbook	Yes	Not mentioned	Acupuncture is an effective and safe treatment for mild cognitive impairment (MCI), but its clinical efficacy is not superior to conventional Western medicine.
Liu et al. [40]	China	6 (330)	RCT	Acupuncture combined with other treatments	Other treatments	MMSE, MQ, MoCA, CDR	Not mentioned	Not mentioned	Not mentioned	Acupuncture and moxibustion have a good effect on the treatment of MCI.
Lu et al. [41]	China	14 (—)	RCT	Acupuncture	Not mentioned	Not mentioned	CONSORT and STRICTA guidelines	Not mentioned	Not mentioned	The reporting quality of RCTs of acupuncture for MCI was moderate to low. The CONSORT statement and STRICTA should be used to standardize reporting of RCTs of acupuncture in the future.
Cao et al. [42]	China	12 (691)	RCT	Acupuncture	No acupuncture therapy	MMSE, MoCA, MBI, WMS	Cochrane Handbook	Yes	Not mentioned	The current clinical evidence is not of sufficient quality for a wider application of acupuncture to be recommended for the treatment of vascular mild cognitive impairment (VMCI).
Hu et al. [43]	China	14 (1052)	RCT	Acupuncture	Various Chinese patent medicines	MMSE	Cochrane Handbook	Yes	Not mentioned	Acupuncture is safe and effective in treating MCI.
Mai and Zheng [44]	China	5 (565)	RCT	Scalp acupuncture	Western medicines alone or other treatments	Total efficacy, MMSE, MoCA	Cochrane Handbook	Yes	Not mentioned	The clinical efficacy of scalp acupuncture in treating MCI was better than that of drug therapy alone, and the efficacy of scalp acupuncture and cognitive training was equivalent.
Deng and Wang [45]	China	5 (568)	RCT or q-RCT	Acupuncture alone or combined with other treatments	Another aggressive treatment	MoCA, MMSE, CDT, WMS	Cochrane Handbook	Yes	Yes	Acupuncture appears effective for amnestic MCI (aMCI) when used as an alternative or adjunctive treatment.
Ting et al. [46]	China	18 (1095)	RCT	Acupuncture-combined Western drug	Same drug as the intervention group	MMSE, ADL	Cochrane Handbook	Yes	Not mentioned	With the present shreds of evidence, acupuncture plus drug is more effective than the drug alone for the treatment of MCI.
Wang [47]	China	25 (1847)	RCT	Acupuncture alone or combined with other therapies; empirical acupuncture	Drug therapy; ordinary acupuncture	Clinical efficacy, MMSE	Not mentioned	Yes	Yes	Acupuncture and moxibustion treatment of MCI is effective and safe.
Li et al. [48]	China	10 (666)	RCT	Acupuncture	No acupuncture therapy	Total efficacy, MMSE, MoCA, ADL	Cochrane Handbook	Yes	Not mentioned	Acupuncture and moxibustion have certain therapeutic advantages in the treatment of MCI.
Kim et al. [49]	Korea	5 (257)	RCT or SR	Electric acupuncture	Western medications	MMSE, MoCA, CMS, CDT	Cochrane Handbook	Yes	Yes	Electroacupuncture was an effective treatment for MCI patients by improving cognitive function.

**Table 11:** Methodological quality assessment of systematic reviews by AMSTAR 2.

	Mao (2011)	Liu (2011)	Xiao (2011)	Cao (2013)	Hu (2014)	Mai (2015)	Min (2016)	Shuai (2016)	Wang (2017)	Li (2018)	Kim (2019)	“Yes” (*n* (%))
Item 1	Y	Y	Y	Y	Y	Y	Y	Y	Y	Y	Y	11 (100.00)
Item 2	N	N	N	N	N	N	N	N	N	N	N	0 (0)
Item 3	N	N	N	N	N	N	N	N	N	N	N	0 (0)
Item 4	Y	Y	Y	PY	Y	PY	Y	Y	PY	Y	PY	7 (63.64)
Item 5	Y	N	Y	Y	Y	N	Y	Y	N	Y	Y	8 (72.73)
Item 6	Y	N	Y	Y	Y	N	Y	Y	N	Y	Y	8 (72.73)
Item 7	Y	N	Y	Y	Y	Y	Y	Y	N	Y	Y	9 (81.82)
Item 8	PY	PY	Y	Y	PY	PY	Y	Y	PY	PY	Y	5 (45.45)
Item 9	Y	N	Y	Y	Y	Y	Y	Y	N	Y	Y	9 (81.82)
Item 10	N	N	N	N	N	N	N	N	N	N	N	0 (0)
Item 11	Y	Y	N	Y	Y	Y	Y	Y	Y	Y	Y	10 (90.91)
Item 12	Y	N	N	N	Y	Y	N	Y	N	N	N	4 (36.36)
Item 13	Y	Y	Y	Y	Y	Y	Y	Y	N	Y	Y	10 (90.91)
Item 14	Y	Y	N	Y	Y	Y	Y	N	N	N	Y	7 (63.64)
Item 15	N	Y	N	N	Y	Y	N	Y	Y	Y	N	6 (54.55)
Item 16	N	N	Y	Y	N	N	Y	Y	N	N	Y	5 (45.45)
“Yes”(*n* (%))	10 (62.50)	6 (37.50)	9 (56.25)	10 (62.50)	11 (68.75)	8 (50.00)	11 (68.75)	12 (75.00)	3 (18.75)	9 (56.25)	10 (62.50)	
Ranking of quality	Very low level	Very low level	Very low level	Very low level	Low level	Very low level	Very low level	Low level	Very low level	Low level	Very low level	

Y: yes; PY: partial yes; and N: no.

**Table 12:** Methodological quality assessment of systematic reviews by AMSTAR 2 (assessor 1).

	Mao 2011	Liu 2011	Xiao 2011	Cao 2013	Hu 2014	Mai 2015	Min 2016	Shuai 2016	Wang 2017	Li 2018	Kim 2019
Item 1	Y	Y	Y	Y	Y	Y	Y	Y	Y	Y	Y
Item 2	N	N	N	N	N	N	N	N	N	N	N
Item 3	N	N	N	N	N	N	N	N	N	N	N
Item 4	Y	Y	Y	PY	Y	PY	Y	Y	PY	Y	PY
Item 5	Y	N	Y	Y	Y	N	Y	Y	N	Y	Y
Item 6	Y	N	Y	Y	Y	N	Y	Y	N	Y	Y
Item 7	Y	N	Y	Y	Y	Y	Y	Y	N	Y	Y
Item 8	PY	PY	Y	Y	Y	Y	Y	Y	Y	Y	Y
Item 9	Y	N	PY	Y	PY	PY	Y	Y	N	Y	Y
Item 10	N	N	N	N	N	N	N	N	N	N	N
Item 11	Y	Y	N	Y	Y	Y	Y	Y	Y	Y	Y
Item 12	Y	N	N	N	Y	Y	N	Y	N	N	N
Item 13	Y	Y	Y	Y	Y	Y	Y	Y	N	Y	Y
Item 14	Y	Y	N	Y	Y	Y	Y	N	N	N	Y
Item 15	N	Y	N	N	Y	Y	N	Y	Y	Y	N
Item 16	N	N	Y	Y	N	N	Y	Y	N	N	Y

Y: yes; PY: partial yes; and N: no.

**Table 13:** Methodological quality assessment of systematic reviews by AMSTAR 2 (assessor 2).

	Mao (2011)	Liu (2011)	Xiao (2011)	Cao (2013)	Hu (2014)	Mai (2015)	Min (2016)	Shuai (2016)	Wang (2017)	Li (2018)	Kim (2019)
Item 1	Y	Y	Y	Y	Y	Y	Y	Y	Y	Y	Y
Item 2	N	N	N	N	N	N	N	N	N	N	N
Item 3	N	N	N	N	N	N	N	N	N	N	N
Item 4	Y	Y	Y	Y	Y	Y	Y	Y	PY	Y	PY
Item 5	Y	N	Y	Y	Y	N	Y	Y	N	Y	Y
Item 6	Y	N	Y	Y	Y	N	Y	Y	N	Y	Y
Item 7	Y	N	Y	Y	Y	Y	Y	Y	N	Y	Y
Item 8	Y	Y	Y	Y	PY	PY	Y	Y	PY	PY	Y
Item 9	Y	N	Y	Y	Y	Y	Y	PY	N	Y	Y
Item 10	N	N	N	N	N	N	N	N	N	N	N
Item 11	Y	Y	N	Y	Y	Y	Y	Y	Y	Y	Y
Item 12	Y	N	N	N	Y	Y	N	Y	N	N	N
Item 13	N	Y	Y	Y	Y	Y	Y	Y	N	Y	Y
Item 14	Y	Y	N	Y	Y	Y	Y	N	N	N	Y
Item 15	N	Y	N	N	Y	Y	N	Y	Y	Y	N
Item 16	N	N	Y	Y	N	N	Y	Y	N	N	Y

Y: yes; PY: partial yes; and N: no.

**Table 14:** Reporting quality assessment of systematic reviews by PRISMA.

	Mao (2011)	Liu (2011)	Xiao (2011)	Cao (2013)	Hu (2014)	Mai (2015)	Min (2016)	Shuai (2016)	Wang (2017)	Li (2018)	Kim (2019)	“Yes” (n (%))
Item 1	Y	Y	N	Y	Y	Y	Y	Y	Y	Y	Y	10 (90.91)
Item 2	PY	PY	PY	PY	PY	PY	PY	PY	PY	PY	PY	0 (0)
Item 3	Y	PY	Y	Y	PY	PY	Y	Y	PY	Y	Y	7 (63.64)
Item 4	PY	PY	PY	PY	PY	PY	PY	PY	PY	PY	PY	0 (0)
Item 5	N	N	N	N	N	N	N	N	N	N	N	0 (0)
Item 6	PY	PY	Y	Y	PY	PY	PY	Y	PY	Y	Y	5 (45.45)
Item 7	Y	Y	Y	Y	Y	Y	Y	Y	Y	Y	Y	11 (100.00)
Item 8	Y	N	N	N	N	N	N	N	N	N	Y	2 (18.18)
Item 9	Y	N	Y	Y	N	N	Y	Y	N	N	N	5 (45.45)
Item 10	Y	N	Y	Y	Y	N	PY	Y	N	PY	Y	6 (54.55)
Item 11	PY	PY	Y	Y	PY	PY	Y	Y	PY	PY	Y	5 (45.45)
Item 12	PY	Y	—	Y	Y	PY	Y	Y	PY	PY	Y	6 (60.00)
Item 13	Y	Y	—	Y	Y	Y	Y	Y	Y	Y	Y	10 (100.00)
Item 14	Y	PY	—	Y	PY	PY	Y	Y	Y	Y	Y	7 (70.00)
Item 15	Y	N	—	Y	Y	N	N	Y	N	N	N	4 (40.00)
Item 16	Y	N	—	N	Y	N	N	Y	N	Y	N	4 (40.00)
Item 17	Y	PY	Y	Y	Y	PY	Y	Y	PY	Y	Y	8 (72.73)
Item 18	PY	PY	PY	Y	PY	Y	PY	Y	PY	PY	Y	4 (36.36)
Item 19	Y	N	—	Y	Y	PY	Y	Y	PY	Y	Y	7 (70.00)
Item 20	Y	Y	—	Y	Y	Y	Y	Y	Y	Y	Y	10 (100.00)
Item 21	Y	Y	—	Y	Y	Y	Y	Y	Y	Y	Y	10 (100.00)
Item 22	N	N	—	N	Y	Y	N	Y	N	Y	N	4 (40.00)
Item 23	N	N	—	N	Y	N	N	Y	N	N	N	2 (20.00)
Item 24	PY	PY	Y	Y	PY	PY	Y	PY	PY	PY	Y	4 (36.36)
Item 25	Y	Y	Y	Y	Y	Y	Y	Y	Y	Y	Y	11 (100.00)
Item 26	Y	Y	Y	Y	Y	Y	Y	Y	Y	Y	Y	11 (100.00)
Item 27	N	N	Y	Y	N	N	N	N	N	N	Y	3 (27.27)
“Yes” (*n* (%))	16 (59.26)	8 (29.63)	11 (64.71)	20 (74.07)	15 (55.56)	9 (33.33)	15 (55.56)	21 (77.78)	8 (29.63)	14 (51.85)	19 (70.37)	

Y: yes; PY: partial yes; N: no; and —: not mentioned.

**Table 15:** Reporting quality assessment of systematic reviews by PRISMA (assessor 1).

	Mao (2011)	Liu (2011)	Xiao (2011)	Cao (2013)	Hu (2014)	Mai (2015)	Min (2016)	Shuai (2016)	Wang (2017)	Li (2018)	Kim (2019)
Item 1	Y	Y	N	Y	Y	Y	Y	Y	Y	Y	Y
Item 2	PY	PY	PY	PY	PY	PY	PY	PY	PY	PY	PY
Item 3	PY	PY	Y	Y	PY	PY	Y	Y	PY	Y	Y
Item 4	PY	PY	PY	PY	PY	PY	PY	Y	PY	PY	PY
Item 5	N	N	N	N	N	N	N	N	N	N	N
Item 6	PY	PY	PY	Y	PY	PY	PY	Y	PY	Y	Y
Item 7	Y	Y	Y	Y	Y	Y	Y	Y	Y	Y	Y
Item 8	Y	N	N	N	N	N	N	N	N	N	Y
Item 9	Y	N	Y	Y	N	N	Y	Y	N	N	N
Item 10	Y	N	Y	Y	Y	N	PY	Y	N	PY	Y
Item 11	PY	PY	Y	Y	PY	PY	Y	Y	PY	PY	Y
Item 12	PY	Y	—	Y	Y	PY	Y	Y	Y	PY	Y
Item 13	Y	Y	—	Y	Y	Y	Y	Y	Y	Y	Y
Item 14	Y	PY	—	Y	PY	PY	Y	Y	Y	Y	Y
Item 15	Y	N	—	Y	Y	N	N	Y	N	N	N
Item 16	Y	N	—	N	Y	N	N	Y	N	Y	N
Item 17	Y	Y	Y	Y	Y	PY	Y	Y	PY	Y	Y
Item 18	PY	PY	PY	Y	PY	Y	PY	Y	PY	PY	Y
Item 19	Y	N	—	Y	Y	PY	Y	Y	PY	Y	Y
Item 20	Y	Y	—	Y	Y	Y	Y	Y	Y	Y	Y
Item 21	Y	Y	—	Y	Y	Y	Y	Y	Y	Y	Y
Item 22	N	N	—	N	Y	Y	N	Y	N	Y	N
Item 23	N	N	—	N	Y	N	N	Y	N	N	N
Item 24	PY	Y	Y	Y	PY	PY	Y	PY	PY	PY	Y
Item 25	Y	Y	Y	Y	Y	Y	Y	Y	Y	Y	Y
Item 26	Y	Y	Y	Y	Y	Y	Y	Y	Y	Y	Y
Item 27	N	N	Y	Y	N	N	N	N	N	N	Y

Y: yes; PY: partial yes; N: no; and —: not mentioned.

**Table 16:** Reporting quality assessment of systematic reviews by PRISMA (assessor 2).

	Mao (2011)	Liu (2011)	Xiao (2011)	Cao (2013)	Hu (2014)	Mai (2015)	Min (2016)	Shuai (2016)	Wang (2017)	Li (2018)	Kim (2019)
Item 1	Y	Y	N	Y	Y	Y	Y	Y	Y	Y	Y
Item 2	PY	PY	PY	PY	PY	PY	PY	PY	PY	PY	PY
Item 3	Y	PY	Y	Y	PY	PY	Y	Y	PY	Y	Y
Item 4	PY	PY	PY	PY	PY	PY	PY	PY	PY	PY	Y
Item 5	N	N	N	N	N	N	N	N	N	N	N
Item 6	PY	PY	Y	Y	PY	PY	PY	Y	PY	PY	Y
Item 7	Y	Y	Y	Y	Y	Y	Y	Y	Y	Y	Y
Item 8	Y	N	N	N	N	N	N	N	N	N	Y
Item 9	Y	N	Y	Y	N	N	Y	Y	N	N	N
Item 10	Y	N	Y	Y	Y	N	Y	Y	N	PY	Y
Item 11	PY	PY	Y	Y	PY	Y	Y	Y	PY	PY	Y
Item 12	PY	Y	—	Y	Y	PY	Y	Y	PY	PY	Y
Item 13	Y	Y	—	Y	Y	Y	Y	Y	Y	Y	Y
Item 14	Y	PY	—	Y	PY	PY	Y	Y	Y	Y	Y
Item 15	Y	N	—	Y	Y	N	N	Y	N	N	N
Item 16	Y	N	—	N	Y	N	N	Y	N	Y	N
Item 17	Y	PY	Y	Y	Y	Y	Y	Y	PY	Y	Y
Item 18	PY	PY	PY	Y	Y	Y	PY	Y	PY	PY	Y
Item 19	Y	N	—	Y	Y	PY	Y	Y	PY	Y	Y
Item 20	Y	Y	—	Y	Y	Y	Y	Y	Y	Y	Y
Item 21	Y	Y	—	Y	Y	Y	Y	Y	Y	Y	Y
Item 22	N	N	—	N	Y	Y	N	Y	N	Y	N
Item 23	N	N	—	N	Y	N	N	Y	N	N	N
Item 24	PY	PY	Y	Y	Y	PY	Y	PY	PY	PY	Y
Item 25	Y	Y	Y	Y	Y	Y	Y	Y	Y	Y	Y
Item 26	Y	Y	Y	Y	Y	Y	Y	Y	Y	Y	Y
Item 27	N	N	Y	Y	N	N	N	N	N	N	Y

Y: yes; PY: partial yes; N: no; and —: not mentioned.

**Table 17:** Quality of evidence in included systematic reviews with GRADE.

Authors (year)	Intervention	Outcomes	Limitations	Inconsistency	Indirectness	Imprecision	Publication bias	Quality of evidence
Mao (2011) [39]	Acupuncture vs. donepezil	MMSE (3)	−1^①^	0	0	−1^④^	−1^⑤^	Very low
	Acupuncture vs. donepezil	MQ (2)	−1^①^	−1^③^	0	−1^④^	−1^⑤^	Very low
Cao et al. (2013) [42]	Acupuncture combined with CFT/donepezil vs. CFT/donepezil	MMSE (6)	−1^①^	−1^③^	0	0	0	Low
		MoCA (1)	−1^①^	−1^③^	0	−1^④^	−1^⑤^	Very low
	Electroacupuncture combined with CFT vs. CFT	MBI (1)	−1^①^	−1^③^	0	−1^④^	−1^⑤^	Very low
	Electroacupuncture combined with nimodipine vs. nimodipine	MBI (1)	−1^①^	−1^③^	0	−1^④^	−1^⑤^	Very low
	Electroacupuncture vs. nimodipine	MBI (1)	−1^①^	−1^③^	0	−1^④^	−1^⑤^	Very low
Hu et al. (2014) [43]	Acupuncture vs. no acupuncture therapy	Effective rate (9)	−1^①^	0	0	0	0	Moderate
	Acupuncture combined with nimodipine vs. nimodipine	MMSE (6)	−1^①^	−1^③^	0	0	0	Low
	Acupuncture combined with donepezil vs. donepezil	MMSE (2)	−1^①^	0	0	−1^④^	−1^⑤^	Very low
Mai and Zheng (2015) [44]	Scalp electroacupuncture vs. nimodipine	Total effective rate (3)	−1^①^	0	0	0	−1^⑤^	Low
		Apparent efficiency (3)	−1^①^	0	0	−1^④^	−1^⑤^	Very low
		MMSE (3)	−1^①^	−1^③^	0	0	−1^⑤^	Very low
	Scalp acupuncture combined with CFT vs. CFT	Total effective rate (2)	−1^①^	0	0	−1^④^	−1^⑤^	Very low
		Apparent efficiency (2)	−1^①^	0	0	−1^④^	−1^⑤^	Very low
		MoCA (2)	−1^①^	0	0	−1^④^	−1^⑤^	Very low
Deng and Wang (2016) [45]	Acupuncture vs. nimodipine	MMSE (3)	−1^①^	0	0	0	−1^⑤^	Low
		Clinical efficacy rate (3)	−1^①^	0	0	0	−1^⑤^	Low
	Acupuncture combined with nimodipine vs. nimodipine	MMSE (2)	−1^①^	0	0	−1^④^	−1^⑤^	Very low
Ting et al. (2016) [46]	Acupuncture combined with Western medicine vs. Western medicine	MMSE (12)	−1^①^	−1^③^	0	0	0	Low
	Acupuncture combined with drug vs. drug	ADL (6)	−1^①^	0	0	0	0	Moderate
Wang (2017) [47]	Acupuncture vs. ?	Effective rate (16)	−1^①^	0	0	0	0	Moderate
	Acupuncture or acupuncture combined with other therapies vs. medicine	MMSE (7)	−1^①^	0	0	0	0	Moderate
	Empirical acupuncture vs. ordinary acupuncture	MMSE (3)	−1^①^	−1^③^	0	0	−1^⑤^	Very low
Li et al. 2018 [48]	Acupuncture vs. no acupuncture therapy	Total effective rate (2)	−1^①^	0	0	−1^④^	−1^⑤^	Very low
		MMSE (9)	−1^①^	−1^③^	0	0	0	Low
		MoCA (5)	−1^①^	−1^③^	0	0	−1^②^	Very low
		ADL (3)	−1^①^	−1^③^	0	0	−1^⑤^	Very low
		CDT(2)	−1^①^	−1^③^	0	−1^④^	−1^⑤^	Very low
Kim et al. 2019 [49]	Electroacupuncture vs. antidementia drugs	MMSE (6)	−1^①^	0	0	0	−1^②^	Low
		MoCA (2)	−1^①^	0	0	−1^④^	−1^⑤^	Very low

① = the design of the experiment with a large bias in random, distributive hiding, or blind. ② = funnel graph asymmetry. ③ = the confidence interval overlaps less, the heterogeneity test *P* is very small, and I^2^ is larger. ④ = the sample size is small, and the confidence interval is wide. ⑤ = fewer studies are included, and there may be greater publication bias. ^?^The original text does not clearly mention what the control group is.

**Table 18:** Quality of evidence in included systematic reviews with GRADE (assessor 1).

Author (year)	Intervention	Outcomes	Limitations	Inconsistency	Indirectness	Imprecision	Publication bias
Mao (2011)	Acupuncture vs. donepezil	MMSE (3)	−1^①^	0	0	−1^④^	−1^⑤^
	Acupuncture vs. donepezil	MQ (2)	−1^①^	0	0	−1^④^	−1^⑤^
Cao (2013)	Acupuncture combined with CFT/donepezil vs. CFT/donepezil	MMSE (6)	−1^①^	−1^③^	0	−1^④^	−1^⑤^
		MoCA (1)	−1^①^	−1^③^	0	−1^④^	−1^⑤^
	Electroacupuncture combined with CFT vs. CFT	MBI (1)	−1^①^	−1^③^	0	−1^④^	−1^⑤^
	Electroacupuncture combined with nimodipine vs. nimodipine	MBI (1)	−1^①^	−1^③^	0	−1^④^	−1^⑤^
	Electroacupuncture vs. nimodipine	MBI (1)	−1^①^	−1^③^	0	−1^④^	−1^⑤^
Hu (2014)	Acupuncture vs. no acupuncture therapy	Effective rate (9)	−1^①^	0	0	0	0
	Acupuncture combined with nimodipine vs. nimodipine	MMSE (6)	−1^①^	−1^③^	0	0	0
	Acupuncture combined with donepezil vs. donepezil	MMSE (2)	−1^①^	0	0	−1^④^	−1^⑤^
Mai (2015)	Scalp electroacupuncture vs. nimodipine	Total effective rate (3)	−1^①^	0	0	0	−1^⑤^
		Apparent efficiency (3)	−1^①^	0	0	−1^④^	−1^⑤^
		MMSE (3)	−1^①^	−1^③^	0	0	−1^⑤^
	Scalp acupuncture combined with CFT vs. CFT	Total effective rate (2)	−1^①^	0	0	−1^④^	−1^⑤^
		Apparent efficiency (2)	−1^①^	0	0	−1^④^	−1^⑤^
		MoCA (2)	−1^①^	−1^③^	0	−1^④^	−1^⑤^
Min (2016)	Acupuncture vs. nimodipine	MMSE (3)	−1^①^	0	0	0	−1^⑤^
		Clinical efficacy rate (3)	−1^①^	0	0	0	−1^⑤^
	Acupuncture combined with nimodipine vs. nimodipine	MMSE (2)	−1^①^	0	0	−1^④^	−1^⑤^
Shuai (2016)	Acupuncture combined with Western medicine vs. Western medicine	MMSE (12)	−1^①^	−1^③^	0	0	0
	Acupuncture combined with drug vs. drug	ADL (6)	−1^①^	0	0	0	0
Wang (2017)	Acupuncture vs. ?	Effective rate (16)	−1^①^	0	0	0	0
	Acupuncture or acupuncture combined with other therapies vs. medicine	MMSE (7)	−1^①^	0	0	0	0
	Empirical acupuncture vs. ordinary acupuncture	MMSE (3)	−1^①^	−1^③^	0	0	−1^⑤^
Li (2018)	Acupuncture vs. no acupuncture therapy	Total effective rate (2)	−1^①^	0	0	−1^④^	−1^⑤^
		MMSE (9)	−1^①^	−1^③^	0	0	0
		MoCA (5)	−1^①^	−1^③^	0	0	−1^②^
		ADL (3)	−1^①^	−1^③^	0	0	−1^⑤^
		CDT(2)	−1^①^	−1^③^	0	−1^④^	−1^⑤^
Kim (2019)	Electroacupuncture vs. antidementia drugs	MMSE (6)	−1^①^	0	0	0	−1^②^
		MoCA (2)	−1^①^	0	0	−1^④^	−1^⑤^

① = the design of the experiment with a large bias in random, distributive hiding, or blind. ② = funnel graph asymmetry. ③ = the confidence interval overlaps less, the heterogeneity test *P* is very small, and I^2^ is larger. ④ = the sample size is small, and the confidence interval is wide. ⑤ = fewer studies are included, and there may be greater publication bias. ^?^The original text does not clearly mention what the control group is.

**Table 19:** Quality of evidence in included systematic reviews with GRADE (assessor 2).

Author (year)	Intervention	Outcomes	Limitations	Inconsistency	Indirectness	Imprecision	Publication bias
Mao (2011)	Acupuncture vs. donepezil	MMSE (3)	−1^①^	0	0	−1^④^	−1^⑤^
	Acupuncture vs. donepezil	MQ (2)	−1^①^	−1^③^	0	−1^④^	−1^⑤^
Cao (2013)	Acupuncture combined with CFT/donepezil vs. CFT/donepezil	MMSE (6)	−1^①^	0	0	0	0
		MoCA (1)	−1^①^	0	0	−1^④^	−1^⑤^
	Electroacupuncture combined with CFT vs. CFT	MBI (1)	−1^①^	0	0	−1^④^	−1^⑤^
	Electroacupuncture combined with nimodipine vs. nimodipine	MBI (1)	−1^①^	0	0	−1^④^	−1^⑤^
	Electroacupuncture vs. nimodipine	MBI (1)	−1^①^	0	0	−1^④^	−1^⑤^
Hu (2014)	Acupuncture vs. no acupuncture therapy	Effective rate (9)	−1^①^	0	0	0	0
	Acupuncture combined with nimodipine vs. nimodipine	MMSE (6)	−1^①^	−1^③^	0	0	0
	Acupuncture combined with donepezil vs. donepezil	MMSE (2)	−1^①^	0	0	−1^④^	−1^⑤^
Mai (2015)	Scalp electroacupuncture vs. nimodipine	Total effective rate (3)	−1^①^	0	0	0	−1^⑤^
		Apparent efficiency (3)	−1^①^	0	0	−1^④^	−1^⑤^
		MMSE (3)	−1^①^	−1^③^	0	0	−1^⑤^
	Scalp acupuncture combined with CFT vs. CFT	Total effective rate (2)	−1^①^	0	0	−1	−1^⑤^
		Apparent efficiency (2)	−1^①^	0	0	−1^④^	−1^⑤^
		MoCA (2)	−1^①^	0	0	0	−1^⑤^
Min (2016)	Acupuncture vs. nimodipine	MMSE (3)	−1^①^	0	0	0	−1^⑤^
		Clinical efficacy rate (3)	−1^①^	0	0	0	−1^⑤^
	Acupuncture combined with nimodipine vs. nimodipine	MMSE (2)	−1^①^	0	0	−1^④^	−1^⑤^
Shuai (2016)	Acupuncture combined with Western medicine vs. Western medicine	MMSE (12)	−1^①^	−1^③^	0	0	0
	Acupuncture combined with drug vs. drug	ADL (6)	−1^①^	0	0	0	0
Wang (2017)	Acupuncture vs. ?	Effective rate (16)	−1^①^	0	0	0	0
	Acupuncture or acupuncture combined with other therapies vs. medicine	MMSE (7)	−1^①^	0	0	0	0
	Empirical acupuncture vs. ordinary acupuncture	MMSE (3)	−1^①^	−1^③^	0	0	−1^⑤^
Li (2018)	Acupuncture vs. no acupuncture therapy	Total effective rate (2)	−1^①^	0	0	−1^④^	−1^⑤^
		MMSE (9)	−1^①^	−1^③^	0	0	0
		MoCA (5)	−1^①^	−1^③^	0	0	−1^②^
		ADL (3)	−1^①^	−1^③^	0	0	−1^⑤^
		CDT (2)	−1^①^	−1^③^	0	−1^④^	−1^⑤^
Kim (2019)	Electroacupuncture vs. antidementia drugs	MMSE (6)	−1^①^	0	0	0	−1^②^
		MoCA (2)	−1^①^	0	0	−1^④^	−1^⑤^

① = the design of the experiment with a large bias in random, distributive hiding, or blind. ② = funnel graph asymmetry. ③ = the confidence interval overlaps less, the heterogeneity test *P* is very small, and I^2^ is larger. ④ = the sample size is small, and the confidence interval is wide. ⑤ = fewer studies are included, and there may be greater publication bias. ^?^The original text does not clearly mention what the control group is.

**Table 20:** Details of MMSE scores in the included literature.

Number	Study	Intervention		MD/OR	95% CI	*P*	Included trials	GRADE rating
		Treatment group	Control group					
1	Mao [39]	Acupuncture	Donepezil	0.63	−0.20–1.46	0.14	2	Very low
2	Cao et al. [42]	Acupuncture combined with CFT/donepezil	CFT/donepezil	1.99	1.09–2.88	<0.0001	6	Low
3	Hu et al. [43]	Acupuncture combined with nimodipine	Nimodipine	1.19	0.67–1.70	<0.00001	6	Low
4		Acupuncture combined with donepezil	Donepezil	0.70	0.24–1.17	0.003	2	Very low
5	Deng and Wang [45]	Acupuncture	Nimodipine	0.99	0.71–1.28	<0.01	3	Low
6		Acupuncture combined with nimodipine	Nimodipine	1.09	0.29–1.8	<0.01	2	Very low
7	Mai and Zheng [44]	Scalp electroacupuncture	Nimodipine	1.33	0.85–1.82	<0.00001	3	Very low
8	Ting et al. [46]	Acupuncture combined with Western medicine	Western medicine	1.73	1.28–2.18	<0.00001	12	Low
9	Wang [47]	Acupuncture or acupuncture combined with other therapies	Medicine	1.54	1.29–1.80	<0.00001	7	Moderate
10		Empirical acupuncture	Ordinary acupuncture	2.98	2.01–3.95	<0.00001	3	Very low
11	Li et al. [48]	Acupuncture	No acupuncture therapy	1.47	1.27–1.66	<0.00001	9	Low
12	Kim et al. [49]	Electroacupuncture	Antidementia drugs	0.65	0.28–1.01	0.0005	6	Low

CFT: cognitive function training; MD: mean difference; and OR: odds ratio.

**Table 21:** Details of MoCA scores in the included literature.

Number	Study	Intervention		MD	95% CI	*P*	Included trials	GRADE rating
		Treatment group	Control group					
1	Cao et al. [42]	Electroacupuncture combined with donepezil	Donepezil	1.37	−0.21–2.95	0.09	1	Very low
2	Mai and Zheng [44]	Scalp acupuncture combined with CFT	CFT	2.12	0.78–3.47	0.002	2	Very low
3	Li et al. [48]	Acupuncture	No acupuncture therapy	0.70	0.33–1.07	0.0002	5	Very low
4	Kim et al. [49]	Electroacupuncture	Antidementia drugs	0.66	0.00–1.32	0.05	2	Very low

**Table 22:** Details of ADL scores in the included literature.

Number	Study	Intervention		MD	95% CI	*P*	Included trials	GRADE rating
		Treatment group	Control group					
1	Ting et al. [46]	Acupuncture plus drug	Drug	5.63	4.40–6.87	<0.001	6	Moderate
2	Li et al. [48]	Acupuncture	No acupuncture therapy	2.00	0.88–3.12	0.0005	3	Very low

**Table 23:** Details of CDT scores in the included literature.

Number	Study	Intervention		MD	95% CI	*P*	Included trials	GRADE rating
		Treatment group	Control group					
1	Li et al. [48]	Acupuncture	No acupuncture therapy	0.63	0.47–0.79	<0.00001	2	Very low

**Table 24:** Details of MBI scores in the included literature.

Number	Study	Intervention		MD	95% CI	*P*	Included trials	GRADE rating
		Treatment group	Control group					
1	Cao et al. [42]	Electroacupuncture combined with CFT	CFT	10.73	6.25–15.21	<0.00001	1	Very low
2		Electroacupuncture combined with nimodipine	Nimodipine	10.57	8.64–12.50	<0.00001	1	Very low
3		Electroacupuncture	Nimodipine	7.99	6.29–9.69	<0.0001	1	Very low

**Table 25:** Details of the effective rate in the included literature.

Number	Study	Intervention		OR/RR	95% CI	*P*	Included trials	GRADE rating
		Treatment group	Control group					
1	Hu et al. [43]	Acupuncture	No acupuncture therapy	2.89	2.10–3. 97	<0. 00001	9	Moderate
2	Deng and Wang [45]	Acupuncture	Nimodipine	1.78	1.19–2.65	<0.01	3	Low
3	Mai and Zheng [44]	Scalp electroacupuncture	Nimodipine	1.53	1.25–1.89	<0.0001	3	Low
4				14.17	3.44–58.44	0.0002	3	Very low
5		Scalp acupuncture combined with CFT	CFT	1.18	0.94–1.47	0.16	2	Very low
6				1.83	0.72–6.64	0.20	2	Very low
7	Wang [47]	Acupuncture	?	1.49	1.36–1.62	<0.0001	16	Moderate
8	Li et al. [48]	Acupuncture	No acupuncture therapy	2.38	1.11–5.11	0.03	2	Very low

^?^The original text does not clearly mention what the control group is.
